# THE ROLE OF LATE-LIFE SOCIAL PARTICIPATION IN THE RISK OF DEMENTIA: A LONGITUDINAL STUDY

**DOI:** 10.1093/geroni/igae098.3236

**Published:** 2024-12-31

**Authors:** Xi Wang, Sita Lujintanon, Pablo Martinez Amezcua, Jennifer Schrack

**Affiliations:** Johns Hopkins University, Baltimore, Maryland, United States; Johns Hopkins University, Baltimore, Maryland, United States; Johns Hopkins University, Baltimore, Maryland, United States; Johns Hopkins Bloomberg School of Public Health, Baltimore, Maryland, United States

## Abstract

Social connectedness may prevent dementia in community-dwelling older adults. However, few studies have long-term follow-up and repeated assessments of social participation. We analyzed the data of adults aged 65 years and over in the United States from the National Health and Aging Trends Study (n = 5,893) from 2012 to 2019. We assessed social connectedness annually with questions about engagement in various activities (“Yes”/”No”), including visiting family/friends, attending religious services, organized activities, and going out for enjoyment. We created a binary composite variable indicating participation in at least one of the activities. Incident dementia was defined using the NHATS dementia algorithm (reported dementia diagnosis, AD8, and neurocognitive test results). We estimated the longitudinal association between social participation and dementia using generalized estimating equation models adjusted for characteristics measured in 2011 (one year before the first exposure assessment), including age, sex, race, marital status, educational attainment, household income, depressive symptoms, and self-rated health. Composite social participation was associated with lower odds of dementia (odds ratio [OR]: 0.41; 95% confidence interval [CI]: 0.31, 0.54). Individually, visiting family/friends (0.53; [0.45, 0.63]), attending religious services (0.64 [0.55; 0.75]), participating in organized activities (0.74; [0.61, 0.89]), and going out for enjoyment (0.38; [0.32, 0.45]) were all associated with lower odds of dementia. Among community-dwelling older adults in the United States, multiple forms of social participation may be targets for dementia prevention interventions or serve as early markers of dementia risk.

